# Effects of eggshell temperature pattern during incubation on primary immune organ development and broiler immune response in later life

**DOI:** 10.1016/j.psj.2020.09.088

**Published:** 2020-10-09

**Authors:** H.J. Wijnen, H. van den Brand, A. Lammers, I.A.M. van Roovert-Reijrink, C.W. van der Pol, B. Kemp, R. Molenaar

**Affiliations:** ∗ Research Department, Hatchtech BV, 3900 AG Veenendaal, The Netherlands; † Adaptation Physiology Group, Department of Animal Sciences, Wageningen University, 6700 AH Wageningen, The Netherlands

**Keywords:** broiler, incubation temperature, embryo development, immune response

## Abstract

Eggshell temperature (**EST**) during incubation greatly affects embryo development, chick quality at hatch, and subsequently various broiler physiological systems. Until now, a constant EST of 37.8°C seems optimal. Data on effects of EST patterns on immune organ development and subsequent broiler immune response are, however, scarce. A higher EST of 38.9°C in week 2 and/or a lower EST of 36.7°C in week 3 of incubation potentially positively affect embryo immune organ development and broiler immune response post hatch. Broiler eggs (n = 468) were incubated at 4 different EST patterns (n = 117 eggs/treatment) from week 2 of incubation onward. Week 1 (embryonic age (**E**)0 < E7) EST was 37.8°C for all eggs. Week 2 (E7 < E14) EST was either 37.8°C (**Control**) or 38.9°C (**Higher**), and week 3 (E14 - /hatch) EST was either Control or 36.7°C (**Lower**). At hatch, histology of bursal follicles and jejunum villi and crypts were determined as well as heterophil to lymphocyte ratio (**H:L)** (n = 49). Posthatch, both sexes were grown in 8 pens/treatment for 6 wk (n = 320). Natural antibodies (**NAb**) were determined at day 14, 22, and slaughter (day 41 or 42) as an indicator of immunocompetence and response to a Newcastle disease (**NCD**) vaccination was determined by antibody levels at day 22 and slaughter (n = 128). Results showed no interaction EST week 2 × EST week 3, except for jejunum histology. Higher EST in week 2 resulted in lower cell density within bursal follicles (*P* = 0.02) and a tendency for lower H:L (*P* = 0.07) at hatch, and higher NCD titers at slaughter (*P* = 0.02) than Control EST. Lower EST in week 3 resulted at hatch in higher cell density within bursal follicles, higher H:L (both *P* < 0.05), and a tendency for a higher posthatch mortality rate than control EST (*P* = 0.10). In conclusion, higher EST in week 2 during incubation may benefit embryonic immune organ development and posthatch broiler immunocompetence, while lower EST in week 3 showed opposite indications.

## Introduction

Infectious diseases in broiler husbandry impair broiler health and welfare and cause major economic losses (Jones et al., 2019). Currently, medicines are frequently used to prevent or cure infectious diseases. However, the use of medicines brings costs and comes with the risk of antimicrobial resistance (**AMR**), which is an increasing worldwide threat to animal and human health (European Food Safety Authority and European Centre for Disease Prevention and Control, 2018). Thus, other strategies rather than the usage of medicines are needed in reducing the incidence of infectious diseases in broiler husbandry.

A well-functioning immune system is essential for broilers to counteract infectious diseases. A promising strategy to enhance a broiler's immunocompetence is by stimulating their immune system already during embryonic development. Embryo development is mainly affected by embryo temperature. Embryo temperature is accurately (±0.2°C) reflected by eggshell temperature (**EST**) during incubation (French, 1997). It has been shown that EST has pronounced effects on embryo body weight, yolk uptake, and development of organs, such as the heart and liver (Lourens et al., 2007; Molenaar et al., 2010; Maatjens et al., 2014; Van der Pol et al., 2014; Nangsuay et al., 2017). Moreover, studies showed that EST can also affect development of primary immune organs. For instance, histology of the bursa, gastrointestinal tract, spleen, and thymus at hatch were affected by high (38.1°C–39.0°C) incubator temperatures (Oznurlu et al., 2010; Liu et al., 2013; Leandro et al., 2017). Therefore, adjustments in EST patterns can potentially improve embryo immune organ development. We hypothesize that improved embryo immune organ development, due to an optimized EST pattern, will affect later life immune functionality. Broilers might, for instance, have a higher production and proliferation of B-cells from their bursa to the peripheral blood. Additionally, invading pathogens can be detected more rapidly by lymphocytes and a rapid onset of phagocytosis and antibody production might occur. Consequently, deleterious effects of infectious diseases on broiler health and performance will be smaller without medical interference because of quick and sufficient responses of the broiler's immune system.

Studies showed that in some avian species, incubation temperature can indeed affect immune response in later life. For instance, in Peking ducks, wood ducks, tree swallows, or quail, incubation temperature manipulations affected humoral or cellular immune response up to 55 d posthatch (Ardia et al., 2010; DuRant et al., 2012; Liu et al., 2013; Burrows et al., 2019; Shanmugasundaram et al., 2019). For chickens, and especially for broilers, studies on the effect of incubation temperature on immune response in later life are limited, and results are inconsistent. Santin et al. (2003) concluded that broiler immune response posthatch was not affected by incubation temperature. In this study, broilers were incubated at a constant incubator temperature of 37.8°C or at 1°C higher or lower from the 14th day of incubation onwards. No difference in antibody titers against Newcastle disease virus (**NCD**) or infectious bursal disease at day 14 or day 35 posthatch were found. Rajkumar et al. (2015) also concluded that broiler immune responses posthatch was not affected by incubation temperature. In their study, broilers were incubated at a constant incubator temperature of 37.8°C or at a 2°C higher incubator temperature during 3 h at day 16, 17, and 18 of incubation. At day 42 posthatch, no differences in NCD antibody titers or swelling reaction in the wattle to Phytohemagglutinin-P injection were found. Increasing incubator temperature by 1°C from day 10 of incubation onward compared with a constant incubator temperature of 37.8°C was found to increase mucin expression and reduce colonization of Salmonella Enteritidis in the cecal content of 10-day-old broilers when inoculated with Salmonella Enteritidis at 2 d posthatch (De Barros Moreira Filho et al., 2015). Somewhat contradictory, the same thermal manipulation treatment by Oznurlu et al. (2010) resulted in lower ACP-ase positive peripheral blood lymphocytes percentage and a higher ratio of heterophils to lymphocytes in broiler chicks at 7 d posthatch.

It appears that incubation temperature can affect broiler immune response in later life, but the EST pattern that result in optimal immune response has probably not been found yet. All studies mentioned in the previous paragraph adjusted incubation temperature treatments based on incubator temperature and not on EST. Incubator temperature differs from EST, and this difference varies between incubator systems because of a difference in capacity to transfer embryonic heat from the egg to the surrounding air (Meijerhof and Van Beek, 1993; Lourens et al., 2005, 2006). This may explain some of the differences found between previous studies. A constant EST of 37.8°C throughout incubation is considered optimal for chick quality at hatch in terms of body length and yolk free body mass (Lourens et al., 2005). However, recently, it was shown that a higher EST of 38.9°C during week 2 of incubation compared with a constant EST of 37.8°C improved embryonic growth rate (Nangsuay et al., 2016) and resulted in a longer chick length at hatch (Wijnen et al., 2020) and improved tibia bone characteristics at slaughter age (Güz et al., 2020). Besides, a lower EST of 36.7°C during week 3 of incubation compared with a constant EST of 37.8°C was shown to result in a higher yolk-free body mass and higher relative weights of the heart, liver, stomach, intestines, spleen (tendency), and bursa (Maatjens et al., 2016a, Maatjens et al., 2016b; Wijnen et al., 2020). These results suggest that a higher EST of 38.9°C during week 2 in combination with a lower EST of 36.7°C during week 3 of incubation may also affect embryo immune organ development and consequently posthatch immune response, but this was not studied. Therefore, the aim of the current study was to investigate whether a higher EST in week 2 and/or a lower EST in week 3 of incubation affect primary immune organ development and posthatch immune response in broilers.

## Material and methods

### Experimental Design

Eggs were incubated in a 2 × 2 factorial arrangement at 37.8°C EST (**Control**) or at 38.9°C EST (**Higher**) during week 2 of incubation and at Control EST or 36.7°C EST (**Lower**) during week 3 of incubation (n = 117 eggs/treatment). The experimental protocol was approved by the Governmental Commission on Animal Experiments, the Hague, The Netherlands; approval number: 2016.W-0087.001. For further details about material and methods, please review Wijnen et al. (2020).

### Incubation

**Figure 1:**
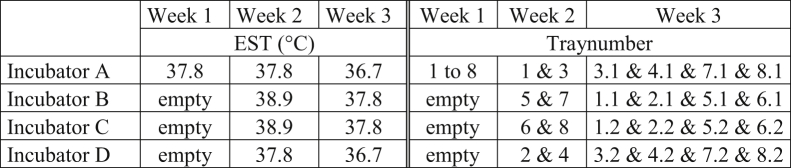
Schematic overview of treatment (eggshell temperature; EST) and eggtrays allocation over 4 different incubators (A to D) during 3 wk of incubation.

Eggs (n = 468) within a 3 g weight range from the average egg weight (63.5 g) of a 44-week-old Ross 308 broiler breeder flock were used. Before the start of incubation, eggs were divided over 8 setter trays (58 or 59 eggs/tray) and warmed linearly in 14 h from storage temperature (20°C) to an EST of 37.8°C. The moment the eggs reached an EST of 37.8°C was considered to be the start of incubation (E0). During week 1 of incubation (E0 up to and including E6), all eggs were incubated in the same incubator at an EST of 37.8°C. During week 2 of incubation (E7 up to and including E13), egg trays were equally divided over 4 incubators (Figure 1). During week 3 of incubation (E8 up to and including hatch), each egg tray was divided over 2 new trays (29 or 30 eggs/tray), and trays were again mixed over 4 incubators according to their treatment (Figure 1). Air temperature within each incubator was continuously adjusted automatically based on the median EST of 4 EST sensors (dry bulb) per incubator (climate respiration chamber type–details provided by Heetkamp et al., 2015). Throughout the incubation period, relative humidity was maintained between 50% and 65, and CO_2_ levels were < 3,500 ppm. Eggs were turned 90° every hour from the start of incubation until embryonic day (**E**) 18.

### Hatch

From E19 h12 onward (468 h of incubation), each incubator was opened every 6 h to mark newly hatched chicks. Six hours after a chick was marked, it was collected from the incubator, feather sexed, and classified either as second-grade chick if any abnormality was observed (e.g., crossed beak, blindness, exposed brains, 4 legs, exposed yolk) or as first-grade chick (all remaining chicks). Every eighth first-grade chick that hatched per treatment was decapitated and dissected. This resulted in dissection of 12 chicks per treatment. All remaining first-grade chicks (n = 337) were provided immediately with feed and water (within 12 h after hatch), and 40 chicks of each sex per treatment were selected for the grow-out period. For details about exact hatch moments, please review Wijnen et al. (2020).

### Grow-Out

Broilers were divided over 2 adjacent houses in 32 floor pens (8 replicate pens/treatment). Within each house, pens were allocated over 4 blocks. Within each block, all 4 treatments were randomly assigned. Each pen contained 5 male and 5 female broilers. Broilers were grown for 6 wk. They were vaccinated against infectious bronchitis at day 1 (Nobilis MA5) and against NCD at day 15 (inactivated Nobilis Newcavac 1000d).

### Data Collection

#### Organ Development

From the dissection chicks at hatch (12 chicks/treatment), the entire bursa was collected as well as approximately 1.5 cm of the first part of the jejunum (right after the distal part of the duodenum). A Swiss roll was made from the jejunum as described by Molenbeek and Ruitenberg (1981). The bursa and intestine samples were fixated in 4% formaldehyde in PBS for 2 d, and thereafter, they were stored in 70% ethanol until processing (approx. 12 mo after hatch). At processing, they were put in paraffin, sliced (jejunum 3 μm and bursa 7 μm), and mounted on a glass microscope slide. Slides were hematoxylin and eosin stained.

**Figure 2:**
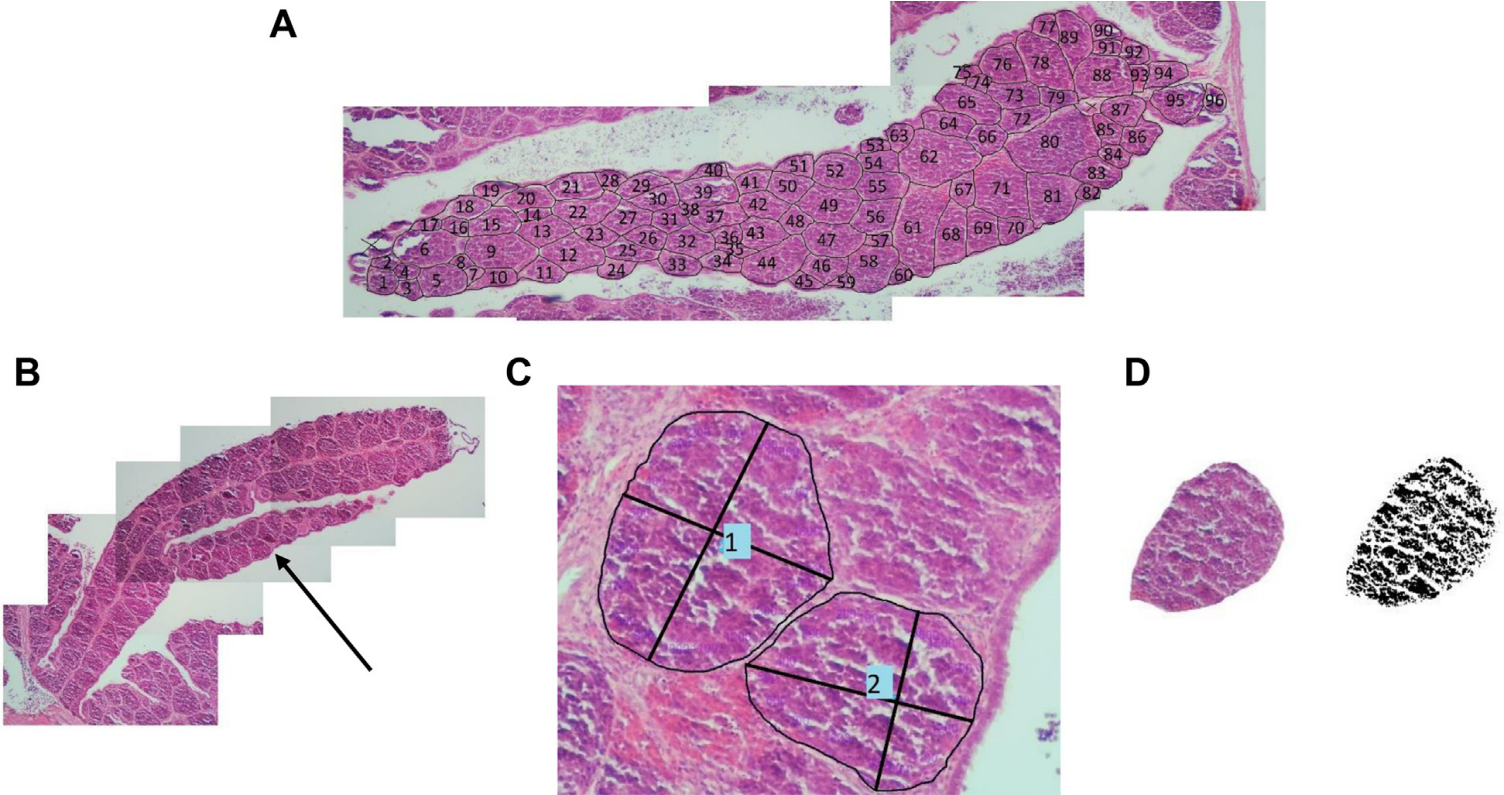
Bursa of Fabricius appearance of broiler chicks at hatch. Follicles within 1 fold were counted (A; n = 96 in this example). Side branches (B; arrow) were included. Follicle length, width, and circumference was measured (C; example 2 follicles). Cell density within follicles was determined by converting the image to black pixels and counting no. of black pixels (D).

One slide per bursa was examined under the microscope (Olympus BX41) for fold and follicle appearance. The longest intact fold inside the bursa slice was photographed with a microscope camera (MC500-W third gen.) on a 4× magnification (Figure 2A). The number of follicles inside this fold was counted with the use of Clip Studio Paint (Version 1.6.2, Figure 2A). Follicles that were damaged (e.g., cut in half, cell leakage, etc.) were not included. If the fold had a side branch in addition to the main branch (Figure 2B), both the main branch and the side branch were included. Occurrence of a side branch was equal for all treatments and seen in 16% of the slices. Thereafter, 10 individual follicles were selected randomly from each fold. These 10 follicles were photographed with a 10× magnification and analyzed. Length and width were measured using the standard straight line tool (Clip Studio Paint, version 1.6.2; Figure 2C). Additionally, circumference was determined by creating a custom ruler to draw a continuous line around the follicles and measuring the length of the line (Figure 2C). Finally, follicle area and cell density within each follicle were determined by converting the follicle into a black and white image (Paint.Net; Figure 2D). To determine the area, all pixels within the circumference were counted. To determine the cell density (%), the number of black pixels were counted and divided by the total number of pixels. For all follicle characteristics, the average of the 10 follicles was calculated, and these averages were used for the statistical analysis.

Photomicrographs of 1 slide per jejunum sample were analyzed on a microscope (Leica DM3000 LED) for villi and crypt appearance and histopathology, using Leica LAS V4.8 software. From each slide, 10 representative villi and associated crypts were randomly chosen. Villi lengths were measured as the distance (in μm) from the villus tip to the villus–crypt junction. Crypt depths were measured as the distance (in μm) from the base of the crypt to the villus–crypt junction (Uni et al., 1995). The averages of these 10 villi and crypts were calculated, and these averages were used in statistical analysis. Histopathological examination was performed by a veterinarian, and the absence or presence was scored of villi fusion, mucosal lymphocyte/plasma cell infiltration, mucosal heterophil infiltration, and enterocyte damage. For each variable, a score 0 was noted in case of absence and 1 in case of presence. All different pathologies were assumed equally bad, and therefore, they were summed to calculate a total histopathology score per slide, ending up in a score of minimum 0 and maximum 4.

#### H:L Ratio

From the dissection chicks at hatch, 1 droplet of blood was collected after decapitation (mixture artery and vein), and a blood smear was made on a microscope slide. A May-Grünwald-Giemsma coloring was applied, and smears were stored at room temperature until they were analyzed by light microscopy. A total of 100 true whole leukocytes (incl. heterophils, lymphocytes, monocytes, basophils, and eosinophils but excl. erythrocytes and thrombocytes) were counted on each slide, and the heterophil to lymphocyte ratio (**H:L**) was calculated.

#### Newcastle Disease Vaccination Response

Antibody response to an inactivated NCD vaccine was determined as a measure of the acquired B-cell reactivity. From the dissection chicks at hatch, blood was collected at hatch via decapitation (mixture of arterial and venous blood). Additionally, from 128 randomly chosen broilers (2 males and 2 females/pen), blood was collected twice; once at 7 d postvaccination (day 22) via the wing vein and once at slaughter via decapitation (mixture arterial and venous blood). Slaughter was either on day 41 or on day 42. Each day, 1 male and 1 female broiler per pen were slaughtered (total of 32 broilers/treatment). Blood was collected in natrium heparinized tubes (Vacuette 4 mL FX, Greiner Bio-One), stored on ice, and plasma was collected after centrifugation at 2,000 × *g* for 10 min. Plasma was stored at −20°C until samples were analyzed for antibody titers for NCD with an ELISA kit (06-01,096-15 IDEXX, Hoofddorp, The Netherlands). Briefly, in 96-well plates coated with NCD antigen the diluted plasma samples were dispensed as well as negative control serum (diluted chicken serum non-reactive to NDV preserved with sodium azide) and positive control serum (diluted chicken anti NCD serum). Well-plates were incubated for 30 min at 20°C, washed with deionized water, and goat anti chicken conjugate (HRPO preserved with gentamicin and proclin) was added. Plates were incubated again for 30 min at 20°C, washed, and TMB substrate was added. Plates were incubated for 15 min at 15°C at 20°C, and stop solution was added.

#### Natural Antibodies

At day 14, 22, and at slaughter (day 41 or 42), blood samples were taken and treated as described above. Plasma samples were analyzed for the level of natural antibodies (**NAb**) through the amount of immunoglobulin binding to keyhole limpet hemocyanin (**KLH**) as described for layer chickens by Berghof et al. (2015).

#### Mortality

Broilers that died were noted daily, and mortality rates were calculated per pen relative to the number of broilers at placement. Four broilers were culled for human reasons (e.g., poor gait) and were excluded from the analysis.

#### Statistical Analyses

All data were analyzed using the statistical software package SAS (Version 9.4, SAS institute, 2010). The variables determined in hatchlings were analyzed using general linear model 1 (Proc Mixed—3-way ANOVA):

[1] $${\text{Y}}_{\text{ijk}}=\mu +\text{ESTwk}2_{\text{i}}+\text{ESTwk}3_{\text{j}}+\text{ESTwk}2\;\times \text{ ESTwk}3_{\text{ij}}+{\text{sex}}_{\text{k}}+{\text{e}}_{\text{ijk}}\text{,}$$

Where, Y_ijk_ = the dependent variable, μ = the overall mean, ESTwk2_i_ = EST in week 2 (_i_ = 38.9 or 37.8°C), ESTwk3_j_ = EST in week 3 (_j_ = 37.8°C or 36.7°C), EST week 2 × EST week 3_ij_ = the interaction between EST week 2 and EST week 3, sex = sex (_k_ = male or female), and e_ijk_ = the error term.

Preliminary statistical analysis did not show significant effects of sex × EST week 2 or sex × ESTwk 3 or sex × EST week 2 × EST week 3 for any of the variables (*P* ≥ 0.07). Therefore, interactions between sex and EST were excluded from the final model.

The hatchling was used as the experimental unit for all hatchling variables. For posthatch variables, pen was used as the experimental unit. For mortality, model 1 was used but without sex. For NCD vaccination response and natural antibodies, measurements were performed on individual broilers but analyzed on pen basis, by extending model 1 with pen (1–32) nested within block (1–8) as a random factor. The NAb titers were measured at 3 moments for the same broiler (day 14, 22, slaughter), and broiler was considered to be the repeated subject. Model 1 was extended with day and the interactions between day and EST week2, day and EST week 3, day and sex. A compound symmetry covariance structure was assumed.

Model assumptions were verified by inspection of residual plots. All data were distributed normally. Tukey adjustments for multiple comparisons were used to compare least square means (LSMeans).

Histopathology score (0–4) of the intestines was analyzed with a generalized linear model (Proc Glimmix), using model 1 and a multinomial cumulative logit link function.

## Results

### Bursa of Fabricius

No interaction between EST week 2 and EST week 3 was found (*P* ≥ 0.10) for any of the bursa characteristics (Table 1). Higher EST in week 2 resulted in a 7% lower cell density within the bursal follicles compared with Control (*P* = 0.024). Lower EST in week 3 resulted in an 8% higher cell density within the bursal follicles compared with Control (*P* = 0.007). Bursa characteristics did not differ between sexes (*P* ≥ 0.37; data not shown).

**Table 1:** Effect of a higher eggshell temperature (EST) of 38.9°C during week 2 of incubation (Higher) and/or a lower EST of 36.7°C during week 3 of incubation (Lower) compared with a control EST of 37.8°C (Control) on histological characteristics of follicles within the Bursa of Fabricius and histology of the jejunum of broiler chicks at hatch.

Treatment	Bursa follicles						Jejunum	
	Follicles/fold^1^ (no.)	Length^1^^,^^2^ (px)	Width^1^^,^^2^ (px)	Circumference^1^^,^^2^ (px)	Area^1^^,^^2^ (px)	Cell Density^1^^,^^2^ (%)	Villi length^3^ (μm)	Crypt depth^3^ (μm)
EST week 2								
Control	70	565	412	1,667	186,238	46^a^	223	97
Higher	76	569	395	1,589	183,912	39^b^	235	92
SEM	5	20	14	53	11,869	2	6	2
EST week 3								
Lower	69	566	389	1,572	176,244	47^a^	225	91
Control	77	568	418	1,684	193,905	39^b^	233	97
SEM	5	20	14	53	11,877	2	6	2
EST week 2 × week 3								
Control × Lower	65	561	381	1,552	167,496	49	207^b^	90^b^
Control × Control	76	569	444	1,782	204,980	43	239^a^	104^a^
Higher × Lower	74	571	398	1,592	184,993	45	242^a^	93^b^
Higher × Control	77	567	393	1,585	182,830	34	227^a,b^	91^b^
SEM	7	28	20	74	16,751	3	9	3
*P*-value								
Week 2	0.45	0.88	0.40	0.29	0.89	0.024	0.21	0.14
Week 3	0.34	0.93	0.15	0.14	0.29	0.007	0.39	0.07
Week 2 × week 3	0.59	0.84	0.10	0.12	0.25	0.44	0.016	0.016

^a-b^Least squares means within a column and factor lacking a common superscript differ (*P* ≤ 0.05).

1 n = 12, n = 11, n = 12, n = 11 for treatments Control × Lower, Control × Control, Higher × Lower, Higher × Control, respectively.

2 Averaged per 10 follicles. Cell density = the % of area within a follicle that is covered with cells.

3 Averaged per 10 villi or crypt. n = 13, n = 12, n = 11, n = 12 for treatments Control × Lower, Control × Control, Higher × Lower, Higher × Control, respectively.

### Jejunum

An interaction was found between EST week 2 and EST week 3 for both villi length and crypt depth (both *P* = 0.016; Table 1). In the Control × Lower EST group villi length was approximately 16% shorter (Δ = 32 μm; *P* = 0.016) and crypt depth approximately 16% shallower (Δ = 14 μm; *P* = 0.016) compared to the other 3 EST treatment groups. Villi length or crypt depth did not differ between sexes (*P* ≥ 0.19; data not shown). Histopathology score did not differ between treatments (*P* ≥ 0.24, data not shown) or between sexes (*P* = 0.86; data not shown).

### H:L Ratio

No interaction between EST week 2 and EST week 3 was found (*P* ≥ 0.30) for the number of heterophils or lymphocytes or H:L ratio at hatch (Table 2). Higher EST in week 2 did not affect the number of heterophils or lymphocytes (*P* > 0.11), but it tended to decrease the H:L ratio by 33% compared to Control (Δ = 2.1 ratio; *P* = 0.07). Lower EST in week 3 resulted in 10% fewer lymphocytes (Δ = 10 cells, *P* = 0.01), 7% more heterophils (Δ = 9 cells, *P* = 0.02), and a 38% higher H:L ratio (Δ = 2.5 ratio, *P* = 0.04) compared with Control. Levels and ratio of heterophil or lymphocytes did not differ between sexes (*P* ≥ 0.30).

**Table 2:** Effect of a higher eggshell temperature (EST) of 38.9°C during week 2 of incubation (Higher) and/or a lower EST of 36.7°C during week 3 of incubation (Lower) compared with a control EST of 37.8°C (Control) on heterophil and lymphocyte occurrence in blood of broiler chicks at hatch.

Treatment	n	Lymphocytes (no./100 cells)	Heterophils (no./100 cells)	H:L ratio
EST week 2				
Control	14	16	81	6.4
Higher	11	22	76	4.3
SEM		2	3	0.8
EST week 3				
Lower	9	14^b^	83^a^	6.6^a^
Control	16	24^a^	74^b^	4.1^b^
SEM		2	3	0.8
EST week 2 × week 3				
Control × Lower	5	12	86	8.3
Control × Control	9	21	76	4.6
Higher × Lower	4	17	81	4.9
Higher × Control	7	27	71	3.6
SEM		3	4	1.1
*P*-value				
Week 2		0.11	0.14	0.07
Week 3		0.010	0.012	0.036
Week 2 × week 3		0.86	0.96	0.30

^a-b^Least squares means within a column and factor lacking a common superscript differ (*P* ≤ 0.05).

Abbreviation: H:L, heterophil to lymphocyte ratio.

### NCD Vaccination Response

No interaction between EST week 2 and EST week 3 was found for NCD titer before vaccination (hatch), at 7 d postvaccination (day 22) and at 27 d postvaccination (Table 3; *P* ≥ 0.58). Higher EST in week 2 had no effect on NCD titer before vaccination and at 7 d postvaccination (*P* ≥ 0.38), but the 27 d postvaccination (at slaughter) NCD titer was higher compared with Control (Δ = 0.2 log10titre; *P* = 0.02). Lower EST in week 3 had no effect on NCD titer before vaccination and at 7 d postvaccination (*P* ≥ 0.15), but the 27 d postvaccination (at slaughter) NCD titer tended to be higher compared with Control (Δ = 0.1 log10titre; *P* = 0.08). Females had higher NCD titers at slaughter age compared with males (2.9 vs 2.6 log10titer for females and males respectively; *P* = 0.02). At hatch and at 7 d postvaccination, NCD titer did not differ between sexes (*P* ≥ 0.28; data not shown).

**Table 3:** Effect of a higher eggshell temperature (EST) of 38.9°C during week 2 of incubation (Higher) and/or a lower EST of 36.7°C during week 3 of incubation (Lower) compared with a control EST of 37.8°C (Control) and sex on Newcastle disease (NCD) titer of broilers at hatch, at day 22 (7 d after NCD vaccination), and slaughter age (day 41 or 42).

NCD titer			
Treatment	Hatch^1^ (log10titer)	Day 22^2^ (log10titer)	Slaughter^2^ (log10titer)
EST week 2			
Control	3.2	1.5	2.6^b^
Higher	3.1	1.7	2.8^a^
SEM	0.1	0.1	0.1
EST week 3			
Lower	3.0	1.5	2.8
Control	3.2	1.7	2.7
SEM	0.1	0.1	0.1
EST week 2 × week 3			
Control × Lower	3.0	1.4	2.7
Control × Control	3.3	1.6	2.6
Higher × Lower	3.0	1.6	2.8
Higher × Control	3.1	1.7	2.8
SEM	0.1	0.2	0.1
*P*-value			
Week 2	0.38	0.41	0.02
Week 3	0.15	0.50	0.08
Week 2 × week 3	0.58	0.79	0.82
Sex	0.28	0.60	0.02

1 n = 13, 12, 12, 12 for treatments Control x Lower, Control× Control, Higher × Lower, Higher × Control, respectively.

2 n = 16/treatment.

### Natural Antibodies

The NAb binding KLH at hatch, day 14, day 22, and slaughter age were neither affected by an interaction between EST in week 2 and EST in week 3 (Table 4; *P* = 0.87) nor by a main effect of EST in week 2 or week 3 (*P* ≥ 0.24). The NAb titers were higher at day 14 compared with day 22 and at slaughter age (*P* < 0.001) but did not differ between day 22 and slaughter age (titer was 3.5, 2.3, 2.4 for day 14, 22, slaughter respectively). Females tended to have higher NAb binding KLH titer at day 22 (*P* = 0.07) compared with males (2.5 vs. 2.0 ± 0.2 respectively; data not shown), but NAb binding KLH titers did not differ between sexes at other ages (*P* ≥ 0.68).

**Table 4:** Effect of a higher eggshell temperature (EST) of 38.9°C during week 2 of incubation (Higher) and/or a lower EST of 36.7°C during week 3 of incubation (Lower) compared to a control EST of 37.8°C (Control) and sex on natural antibodies (NAb) titer against keyhole limpet hemocyanin of broilers at hatch, day 14, 22, and slaughter age (day 41 or 42).

NAb titer				
Treatment	Hatch^1^	Day 14^2^	Day 22^2^	Slaughter^2^
EST week 2				
Control	9.2	3.5	2.3	2.4
Higher	9.6	3.6	2.2	2.5
SEM	0.5	0.2	0.2	0.2
EST week 3				
Lower	9.0	3.4	2.2	2.4
Control	9.8	3.7	2.4	2.4
SEM	0.5	0.2	0.2	0.2
EST week 2 × week 3				
Control × Lower	8.7	3.4	2.3	2.4
Control × Control	9.7	3.6	2.4	2.3
Higher × Lower	9.3	3.5	2.2	2.5
Higher × Control	10.0	3.7	2.3	2.5
SEM	0.7	0.2	0.2	0.2
*P*-value				
Week 2	0.54		0.74	
Week 3	0.24		0.38	
Week 2 × week 3	0.87		0.93	
Sex	0.70		0.76	
Week 2 × day	n.a.		0.80	
week 3 × day	n.a.		0.74	
Week 2 × week 3 × day	n.a.		0.97	
Day	n.a.		<0.001	
Sex × day	n.a.		0.08	

1 n = 13, 12, 12, 12 chicks for treatments Control x Lower, Control × Control, Higher × Lower, Higher × Control, respectively.

2 n = 16 pens/treatment.

### Mortality

Mortality rate was neither affected by an interaction between EST in week 2 and EST in week 3 (*P* = 0.73; data not shown) nor by a main effect of EST in week 2 (*P* = 0.73). Lower EST in week 3 tended to increase mortality rate (Δ = 3.1%; *P* = 0.10) compared with Control.

## Discussion

The aim of this study was to investigate whether a higher EST of 38.9°C in week 2 in combination with a lower EST of 36.7°C in week 3 of incubation affects primary immune organ development and posthatch immune response in broilers compared with a constant EST of 37.8°C. Although no interaction was found between EST week 2 and EST week 3 for the majority of variables that were measured in the current study, some effects were found from either a higher EST in week 2 or a lower EST in week 3.

A higher EST of 38.9°C in week 2 tended to decrease the peripheral H:L ratio at hatch compared to a constant EST of 37.8°C. The H:L ratio has been shown to be associated with parasitic infection, because lymphocytes migrate to the site of infection (Davis et al., 2004; Lobato et al., 2005). However, in the current experiment any effect of an infection on the H:L ratio was not very likely, because the H:L ratio was detected in blood that was collected immediately after removing chicks from the incubator (within 12 h after hatch). Moreover, a parasitic infection would also have caused an increase in monocyte and eosinophil numbers (Jain, 1986; Davis et al., 2004), but no eosinophils were found in any of the blood smears and only 2.2% of all leukocytes were monocytes (data not shown). The H:L ratio has also been used to indicate to what extent a chicken experienced physical or physiological stress (Gross and Siegel, 1983; Beuving et al., 1989; Altan et al., 2000; Davis et al., 2000; Elston et al., 2000; Onbaşilar and Aksoy, 2005; Nicol et al., 2006), because a stressor results in higher corticosterone levels and corticosterone lowers the number of peripheral lymphocytes and increases the number of heterophils (Shapiro and Schechtman, 1949; Weller and Schechtman, 1949; Bannister, 1951; Glick, 1958; Wolford and Ringer, 1962; Bishop et al., 1968; Dhabhar, 2002). Other studies found that thermal manipulations during incubation can cause heat or cold stress to the embryo (Givisiez et al., 2001; Moraes et al., 2002). However, it is not likely that the difference in EST during week 2 (E7 to E14) from the current experiment induced embryonic stress and caused the difference in H:L ratio at hatch moment. Stress alters corticosterone levels via the hypothalamic–pituitary–adrenal axis, and this axis seems functional only from E13 or E14 onward (Case, 1952; Woods et al., 1971; Scott et al., 1981; McIlhone, 2011). Also, heterophils from neonatal chicks are naïve and inefficient (Zulkifli and Siegel, 1994; Lowry et al., 1997; Wells et al., 1998; Genovese et al., 2000) and might therefore be unreactive to corticosterone. Alternatively, the lower H:L ratio at hatch after a higher EST of 38.9°C in week 2 of incubation might be the result of a difference in proliferation of lymphocytes. Regardless of treatment group, the H:L ratio at hatch moment was high (≥3.6) in the current experiment. Other studies demonstrated that a high H:L ratio is normal in newly hatched chicks and that this ratio decreases during the first days of life (Lucas and Jamroz, 1961; Burton and Harrison, 1969; Zulkifli and Siegel, 1994; Maxwell et al., 1997; Gonzales et al., 2003; Oznurlu et al., 2010). The H:L ratio probably decreases during early life because lymphocytes start to migrate from maturation sites, such as the bursa, to the periphery during these days (Lucas and Jamroz, 1961; Cain et al., 1969; Jankovic et al., 1975; Lasilla, 1989). The lower H:L ratio in combination with a lower cell density within bursal follicles at hatch when EST in week 2 was 38.9°C could indicate that more lymphocytes proliferated already from the bursa to the peripheral blood. There are indications that a lower H:L ratio at hatch is predictive of improved later life production and reproduction traits (Al-Murrani et al., 2006) and, if this lower H:L ratio is consistent over time, of higher immune response against a Salmonella typhimurium infection (Al-Murrani et al., 2002). On the one hand, broilers in the current study incubated at a higher EST of 38.9°C in week 2 had higher NCD antibodies at slaughter and therefore the lower H:L ratio that was found at hatch in this group might indeed be predictive for a higher immune response in later life. On the other hand, a lower mortality rate and higher NAb titer would have strengthen the results further, but no effect was found of a higher EST of 38.9°C in week 2 on NAb titer or mortality rate. In layer-type chicken, NAb levels binding to KLH have been found to negatively correlate with survival (Star et al., 2007; Sun et al., 2011; Wondmeneh et al., 2015) and mortality and morbidity levels after an avian pathogenic *Escherichia coli* challenge (Berghof et al., 2019). However, immune response differs between broiler and layer-type chicken (Koenen et al., 2002; Simon et al., 2016) and therefore, the correlation between NAb titer binding KLH and immunocompetence that has been shown in layers, might not be similar for broilers.

A lower EST of 36.7°C in week 3 was expected to improve embryo development compared with a constant EST of 37.8°C. Maatjens et al. (2016a) showed that a lower EST of 36.7°C from E15 onward resulted in chicks with a higher yolk-free body mass and a higher relative heart weight at hatch than an EST of 37.8°C. In the current experiment, no differences in yolk-free body mass were found when EST was lowered to 36.7°C in week 3 compared with a constant EST of 37.8°C (Wijnen et al., 2020). Moreover, the current study found some indications that the lower temperature in the last week of incubation impaired organ maturation instead of improving embryo development. For instance, a lower EST of 36.7°C in week 3 resulted in a higher cell density within bursal follicles and a lower number of peripheral lymphocytes in ratio to heterophils, which probably indicates impaired proliferation of B-cells from the bursa as discussed above. Also, jejunum villi length at hatch was shorter after a lower EST of 36.7°C in week 3 compared to a constant EST of 37.8°C. Intestinal villi grow rapidly during the last 5 d before hatch (Grey, 1972; Uni et al., 1995, 2003). Lowering the EST during these last days of incubation in the current experiment might have slowed down villi growth. Chicken embryos act mainly as poikilotherms (French, 1997) and therefore a lower EST than 37.8°C could have slowed down embryo metabolism and consequently growth of organs such as the intestines. Studies evaluating the effect of a lower EST than 37.8°C during late incubation on intestinal morphology at hatch are lacking. Studies evaluating the opposite effect, an EST higher than 37.8°C during late incubation, did not find the opposite effect of longer intestinal villi at hatch (Barri et al., 2001). In fact, it seems that higher EST during late incubation impairs intestinal development instead (Wineland et al., 2006). However, the finding that a higher EST than 37.8°C during late incubation impairs intestinal villi length does not rule out the possibility that a lower EST than 37.8°C in this period slowed down intestinal villi growth. During late incubation, the amount of oxygen available to the embryo is limited by the conductance of the eggshell (Romijn and Roos, 1938; Rahn et al., 1974; Visschedijk et al., 1985). Raising temperature during this period likely results in an imbalance between metabolic rate and oxygen availability and consequently in impaired organ development (Lourens et al., 2007), while lowering temperature during this period cannot cause this imbalance. Whether the lower EST of 36.7°C during the last week of incubation has consequences for broiler immune response in later life remains unclear from the current results. After hatch, no significant differences in NCD titers and NAb titers were found between a lower EST of 36.7°C in week 3 and a constant EST of 37.8°C, suggesting that there were no differences in immune competence in later life. Nevertheless, a tendency (*P* = 0.10) for a higher overall mortality in the 36.7°C EST treatment shows that overall broilers health might have been worse in this group. The possible difference in broiler mortality, which mainly occurred at the end of the grow-out period, might not be related to a difference in immune response, or it might not be related to a difference in NCD vaccination response and NAb titers at the time points in the current study, but more related to other physiological differences such as thermoregulatory competences.

To predict effects of a higher EST of 38.9°C in week 2 or a lower EST of 36.7°C in week 3 on later life immune response more research is suggested in challenging environments. The current experiment was conducted in a research facility with accurate and optimal management procedures and at low animal stocking densities. It is likely that the immune system was therefore only activated to a relatively low level. Any possible difference in functioning of the immune system might not have been revealed or detected. A follow-up study in which the response of broilers to a validated challenge model can be measured accurately at very specific time points might reveal whether incubation temperature patterns have beneficial or detrimental effects for later life broiler immune response or whether it does not make a difference compared with a constant EST of 37.8°C.

In conclusion, immune organ development at hatch and immune response in later life does not seem to be influenced by the incubation temperature pattern with a higher EST of 38.9°C in week 2 and a lower EST of 36.7°C in week 3. Nevertheless, a higher EST of 38.9°C in week 2 as well as a lower EST of 36.7°C in week 3 both affected immune organ development at hatch and immune response posthatch compared with a constant EST of 37.8°C. A higher EST of 38.9°C in week 2 showed some indications that immune organ development and immune response were perhaps positively affected, whereas a lower EST of 36.7°C in week 3 showed some indications that immune organ development and immune response were negatively affected. Yet, only some immune parameters were studied and only during fixed time points, so more research is needed to confirm or disprove these indication and to study whether incubation temperature patterns affect primary immune organ development and posthatch broiler immune response.
